# Cognitive Assessment with CognoSpeak: Exploring Age‐Related Variations in Attitudes Towards Artificial Intelligence

**DOI:** 10.1002/alz70856_101023

**Published:** 2025-12-25

**Authors:** Xinzhu Cao, Sharon Abrahams, Daniel J. Blackburn

**Affiliations:** ^1^ University of Edinburgh, Edinburgh, United Kingdom; ^2^ The Euan MacDonald Centre, Edinburgh, United Kingdom; ^3^ University of Sheffield, Sheffield, South Yorkshire, United Kingdom

## Abstract

**Background:**

This study investigates the age‐related differences in attitudes towards artificial intelligence (AI) and a specific AI‐based cognitive assessment tool, CognoSpeak, using a mixed‐methods approach.

**Method:**

This study investigated age‐related differences in attitudes towards artificial intelligence (AI) and a specific AI‐based cognitive assessment tool, CognoSpeak, using a mixed‐methods approach. 95 participants completed an online survey, while 20 participants engaged in semi‐ structured interviews. Participants were divided into younger adults (18‐54 years) and older adults (55+ years).

**Results:**

Results from the linear model indicated that there were no significant differences between the two groups in their general attitudes towards AI (total score: b = 3.11, SE = 3.41, t(83) = 0.91, *p* =  0.364) or their attitudes towards CognoSpeak (b = ‐ 2.46, SE = 2.39, t(83) = ‐1.03, *p* =  0.306). However, correlation analysis revealed a moderate positive correlation between general AI attitudes and attitudes toward CognoSpeak (*r* = 0.47, *p* <0.001), suggesting that individuals with more positive general attitudes toward AI also tend to have more favorable views of CognoSpeak. Qualitative analysis of the interviews revealed three overarching themes; Assessment Process ;Interaction with Users The Impact of General Attitudes Towards AI; Across both age groups, participants generally found the assessment process of CognoSpeak to be straightforward and user‐friendly. However, older adults were more likely to express a preference for interacting with real doctors, citing concerns about the lack of empathy and the inability of AI to recognize non‐verbal cues. In contrast, younger participants were more inclined to favour healthcare AI products like CognoSpeak, highlighting its convenience, time‐saving benefits, and the ability to discuss personal issues without fear of judgment. Despite these differences, both age groups acknowledged the potential advantages of AI in healthcare, such as increased accessibility and efficiency.

**Conclusion:**

**T**his study highlights the nuanced attitudes towards AI in healthcare across different age groups, emphasizing the importance of user experience and the need to address specific concerns to enhance the acceptability and effectiveness of AI‐based tools like CognoSpeak. Future research should focus on exploring the underlying reasons for these differences and developing strategies to improve the integration of AI into healthcare services.

